# Preliminary Psychometric Evaluation of the Mental Health Literacy Questionnaire for Young Adults (MHLq-YA) Among Greek University Students

**DOI:** 10.3390/healthcare14203366

**Published:** 2026-10-09

**Authors:** Argyroula Kalaitzaki, Areti-Christina Efthymiou, Michael Rovithis, Eirini Makraki, Argiro Androulidaki, Luisa Campos, Pedro Dias

**Affiliations:** 1 Social Work Department, Hellenic Mediterranean University, 71410 Heraklion, Greece; aefthymiou@hmu.gr; 2 Laboratory of Interdisciplinary Approaches for the Enhancement of Quality of Life, School of Health Sciences, 71410 Heraklion, Greece; rovithis@hmu.gr; 3 Hellenic Scientific Society for Health Literacy, 10431 Athens, Greece; 4 Department of Business Administration & Tourism, Hellenic Mediterranean University, 71410 Heraklion, Greece; 5 Department of Nursing, University of Peloponnese, 22100 Tripolis, Greece; renacrete@hotmail.com; 6 Human Resources Development Directorate, 7th Health Region of Crete, 71500 Heraklion, Greece; 7 Research Centre for Human Development, Universidade Católica Portuguesa, 4169-005 Porto, Portugal; mcampos@ucp.pt (L.C.); pmbdias@ucp.pt (P.D.); 8 Department of Psychology, University of the Azores, 9500-321 Ponta Delgada, Portugal

**Keywords:** mental health literacy, MHLq-YA, validation, psychometric properties, Greece, factor analysis

## Abstract

Background/Objectives: The rising yet underdiagnosed prevalence of mental disorders among young adults, due to stigma, limited knowledge, and reduced help-seeking, underscores the need for culturally validated tools to measure and enhance mental health literacy. This cross-sectional study aimed to validate the 29-item Mental Health Literacy Questionnaire for Young Adults (MHLq-YA) within the Greek context. Methods: A sample of 211 university students (mean age = 20.72 years) was recruited via online convenience and snowball sampling and completed the MHLq-YA, the Mental Health Promoting Knowledge scale (MHPK-10), and the Mental Help-Seeking Intention Scale (MHSIS). Results: Confirmatory Factor Analysis revealed that the originally proposed four-factor structure did not adequately fit the data. An Exploratory Factor Analysis (EFA) followed to identify a structure that better represented the Greek sample. After removing four items due to low factor loadings and poor conceptual alignment, EFA supported a three-factor solution of 25 items, explaining 41.93% of the variance with satisfactory internal consistency: “Knowledge about mental health issues and health behaviors” (α = 0.89), “Erroneous beliefs/stereotypes” (α = 0.70), and “Professional help-seeking behaviors” (α = 0.80). Known-group comparisons showed that mental health literacy scores differed by education and exposure to mental health issues. Inter-scale correlations ranged from low to moderate, and Heterotrait–monotrait ratios (HTMT < 0.85) supported discriminant validity. Moderately high positive associations between the MHSIS and “Professional help-seeking behaviors” (0.56) provided evidence of convergent validity. Conclusions: Overall, the 25-item MHLq-YA demonstrated preliminary construct validity and satisfactory reliability, offering a provisionally culturally adapted instrument for assessing mental health literacy in terms of knowledge, erroneous beliefs, and professional help-seeking behaviors.

## 1. Introduction

### 1.1. Need for Mental Health Literacy

Mental disorders are currently one of the leading causes of disability, substantially contributing to the global disease burden [1]. Among adolescents and young adults (15–39 years old), the global prevalence and burden of mental illness have increased significantly since 1990, representing a major public health concern [2,3]. A sharp increase in stress, anxiety, and depressive symptoms in 2020–21 seems to have been attributed to the pandemic [2,4,5].

This global increase in the prevalence of mental health problems necessitates prevention and intervention measures. However, several interrelated barriers, such as stigma and a lack of knowledge regarding the early detection of signs and symptoms and access to mental healthcare, prevent individuals from seeking help and hinder the effective prevention and management of mental health issues in general [6,7,8]. A thorough understanding of the symptoms, risk factors, and treatment modalities to enable individuals to engage proactively in mental health–promoting behaviors and make informed decisions [9,10,11] requires a substantial level of health-related skills. These skills can be accommodated by a relevant concept and a key strategy for promoting mental well-being and preventing mental health issues, known as “mental health literacy” (MHL).

### 1.2. The Concept of Mental Health Literacy (MHL)

The term Mental Health Literacy (MHL) was originally introduced by Jorm and colleagues [12] (p. 182), who defined it as “knowledge and beliefs about mental disorders which aid their recognition, management, or prevention”. A later refinement of the concept incorporated first-aid-related competencies, namely the ability to support individuals experiencing mental health issues [13]. Consequently, MHL encompasses not only cognitive understanding but also beliefs and attitudes that shape mental health behaviors [1]. The five components of MHL are therefore [13]: (1) knowledge of how to prevent mental disorders; (2) recognition of when a disorder is developing; (3) knowledge of help-seeking options and available treatments; (4) knowledge of effective self-help strategies for milder problems; and (5) first aid skills to support individuals developing a mental disorder or experiencing a mental health crisis. High MHL has been associated with improved health outcomes, such as reduced depression, anxiety, and stress [14,15], reduced stigma, enhanced willingness to interact with people experiencing mental health issues, and an increased preparedness to seek professional support [16,17,18,19,20]. Conversely, low MHL has been linked to depression, anxiety, and widespread misconceptions about mental illness, leading to public, personal, and associative stigma. These stigmatizing beliefs can lead to discrimination, reduce help-seeking behaviors, and result in poorer mental health outcomes [21,22]. Despite its importance, studies in Europe suggest that more than half of the adult population has inadequate health literacy, which affects their ability to maintain good health [23]. This suggests the urgent necessity to enhance public MHL globally. To this end, appropriate instruments are required to evaluate public MHL levels, determine the effectiveness of implemented actions to close knowledge gaps, and enable cross-cultural comparisons.

### 1.3. Assessment of Mental Health Literacy

Research on MHL, which gained significant interest after 2000, has led to numerous assessment tools [1,6]. However, most of these tools focus on limited dimensions such as knowledge or stigma [24] or address specific disorders such as schizophrenia or depression [1]. Furthermore, researchers often overlook the cultural diversity that can influence perceptions of mental illness and health-seeking behaviors [1,24]. Given these limitations, only a few instruments have attempted to provide a more comprehensive assessment of the multiple components of MHL but have been developed nationally for specific cultural contexts, such as the United States (e.g., the three-factor, 26-item MHL) [25] and Australia (e.g., the 35-item Mental Health Literacy Scale) [26].

The Mental Health Literacy questionnaire—Young Adult Form (MHLq-YA) [27] was created and validated among university students by Portuguese researchers and is an adaptation of the original Mental Health Literacy Questionnaire-Young People Form (MHLq-YP) [28]. The MHLq-YA comprises 29 items rated on a five-point Likert scale from 1 (Strongly Disagree) to 5 (Strongly Agree), allocated into four subscales: (1) knowledge of mental health problems, (2) erroneous beliefs/stereotypes, (3) first aid skills and help-seeking behavior, and (4) self-help strategies. It has demonstrated robust psychometric properties in Portuguese. Although the subsequent 16-item shortened version (MHLq-SVa) has been translated and validated in several languages (i.e., American English, Brazilian Portuguese, European Portuguese, Chinese, German, Greek, Hindi, Indonesian, Lithuanian, Malay, Nepali, Nigerian, Polish, Serbian, Thai, Turkish, and Urdu) [1,6,29], only a few translations of the original, longer version are currently available, such as in Chichewa for use in Malawi [30], Turkish [31], Brazilian Portuguese [32], and none in Greek.

### 1.4. Research Objectives

Developing contextually appropriate, validated instruments that thoroughly and comprehensively assess all dimensions of MHL is essential for cross-national validation and comparison. Furthermore, longer versions of instruments tend to be more valid and reliable, reduce measurement error, and provide better content coverage, capturing more nuances of the construct, compared to shorter forms [33,34]. Based on these arguments, this study aimed to preliminarily validate the longer Mental Health Literacy Questionnaire for Young Adults (MHLq-YA) in Greek university students.

## 2. Materials and Methods

### 2.1. Study Design and Procedure

This cross-sectional study was conducted in 2023 in Greece as part of a broader Multicultural Assessment of Mental Health Literacy, a multicenter consortium involving researchers from more than 15 countries worldwide who administer multiple versions of the MHLq (i.e., MHLq-YP, MHLq-YA, and MHLq-SVa) [1,6]. Procedures for translating and administering the MHLq were standardized and documented in an agreement signed by the principal investigator (L.C.) of the consortium and all site investigators.

Participants were recruited online using convenience and snowball sampling methods, with participants encouraged to invite members of their own social networks to take part. The two recruitment methods were combined to increase reach and access to eligible participants who were not easily reachable through the investigators’ contacts. Advertisements were published on social media platforms and in community groups (e.g., student, health, and university-affiliated groups), as well as on various webpages. The questionnaire was administered via Google Forms to ensure accessibility and ease of participation. The informed consent form was included on the introductory page of the online questionnaire, and participants were required to indicate their agreement before proceeding. This page included key information about the study’s aims and procedures, the voluntary nature of participation, and data-handling practices (i.e., stored securely and accessed only by the research team).

The study was reviewed and approved by the appropriate institutional research ethics committee (Νο 130/25-10-2022), ensuring that all procedures met established ethical standards.

### 2.2. Participants

A total of 211 university students from all higher educational institutions in Crete, Greece, participated in the study with a mean age of 20.72 years (SD 1.72, min. 19 max. 25). Students were residing in Crete for their university studies but originated from any region of Greece. Most of them were women (*n* = 147, 69.7%), single (*n* = 147, 69.7%), of Greek nationality (*n* = 209, 99.1%), living in an urban region (*n* = 151, 71.6%), and holding a bachelor’s degree (*n* = 201, 95.3%). Being mostly women and undergraduate students are typical demographic features in other validation studies [27,29,31]. Most participants came from Health and Humanities departments (*n* = 119, 56.4%). Almost half of the participants reported not having a mental health issue themselves (*n* = 126, 59.7%) or having a relative (*n* = 91, 43.1%) or knowing someone (*n* = 103, 48.8%) with a mental health issue. Over half of the participants had no knowledge of mental health issues, as they had not attended a relevant seminar (*n* = 127, 60.2%). Participants’ descriptive statistics are presented in Table 1.

No formal a priori power analysis was performed. In line with previous validation studies [29], sample size adequacy was instead evaluated a priori using established subject-to-item ratio guidelines (a minimum of 5–10 participants per item) [35]. The present sample of 211 participants corresponds to a ratio of approximately 7.3 participants per item (211/29), which meets this benchmark. This a priori justification was subsequently supported post hoc by the Kaiser–Meyer–Olkin (KMO) minimum threshold of 0.60 for sampling adequacy [36].

### 2.3. Measures

Mental Health Literacy Questionnaire for young adults (MHLq-YA). Dias and colleagues [27] adapted the questionnaire for young adults from the Mental Health Literacy questionnaire of young people aged 12–14 years (MHLq-YP) [28]. The development of the MHLq was grounded in Jorm’s conceptualization of Mental Health Literacy [13]. The MHLq-YA comprises 29 items rated on a 5-point Likert scale (1 = strongly disagree to 5 = strongly agree) and includes four subscales: (1) Knowledge of mental health problems (11 items; e.g., “A person with depression feels very miserable.”; “People with schizophrenia usually have delusions.”), (2) Erroneous beliefs/stereotypes (eight items, six of which were reverse-scored: items 6, 10, 13, 15, 23, and 27; e.g., “Mental disorders don’t affect people’s behaviors”; “People with mental disorders belong to low-income countries.”), (3) Help-seeking and first aid skills (six items; e.g., “If I had a mental disorder, I would seek my relatives’ help.”; “If someone close to me had a mental disorder, I would encourage her/him to look for a psychologist.”), and (4) Self-help strategies (four items; e.g., “Physical exercise contributes to good mental health.”; “Sleeping well contributes to good mental health.”). The MHLq-YA’s total score is calculated by summing all item responses (min = 29 to max = 145). The MHLq-YA has demonstrated good internal consistency, with a Cronbach α of 0.84 for the total scale and α values ranging from 0.60 to 0.74 for the subscales [27].

Mental Health Promoting Knowledge scale (MHPK-10). The Mental Health Promoting Knowledge scale is a unidimensional questionnaire with high internal consistency (McDonald’s omega = 0.84) and good construct validity [37]. It was originally developed in Norway using a sample of upper secondary school students, with the aim of assessing knowledge regarding factors that promote positive mental health [37]. The MHPK consists of 10 items rated on a 5-point Likert scale (1 = strongly disagree to 5 = strongly agree), and the total score is calculated as the mean of the 10 items, with higher scores indicating higher mental health knowledge. The original scale has demonstrated a strong positive correlation with health literacy scales. The scale has been validated in three countries: Portugal, Turkey, and China. As there was no Greek validation available, the questionnaire was translated and back-translated by bilingual healthcare researchers for use in the present study. Internal consistency for the Greek version was satisfactory (Cronbach α = 0.89).

Mental Help-Seeking Intention Scale (MHSIS). MHSIS is a unidimensional measure designed to assess individuals’ intention to seek help from a mental health professional if they had a mental health concern [38]. It consists of 3 items rated on a 7-point Likert scale (1 = strongly disagree to 7 = strongly agree), and the total score is calculated as the mean of the 3 items, with higher scores indicating a stronger intention to seek help from a mental health professional. The scale has demonstrated strong psychometric properties, including high internal consistency and good construct validity through associations with attitudes toward help-seeking and prior help-seeking behavior [38]. For the present study, the scale was translated and back-translated into Greek by bilingual clinical psychology researchers. Internal consistency for the Greek version was satisfactory (Cronbach’s α = 0.77), supporting its reliability for use with Greek university students. Finally, participants reported basic demographic information (Table 1).

### 2.4. Translation and Adaptation

The questionnaire was translated into Greek in accordance with the guidelines provided by the primary investigators of the multicenter survey following Erkut’s procedures [39]. The process involved: (1) forward translation from English by a bilingual healthcare professional; (2) back-translation by an independent bilingual healthcare professional; (3) pilot testing of item comprehension with four students using think-aloud procedures; (4) semantic comparison of the forward and back-translated versions; (5) expert review by two specialists in mental health literacy; and (6) final evaluation of the translated version by the Portuguese research team.

During the think-aloud pilot testing, participants were asked to express their understanding of each item, identify unclear wording, and comment on cultural relevance. Feedback indicated that most items were easily understood, with minor phrasing of two or three items, which were incorporated into the final Greek version. Similarly, the expert review confirmed that the translated items were semantically and conceptually equivalent to the original English version, and no major concerns regarding cultural relevance or clarity were highlighted. Minor adjustments to improve fluency in Greek were made, but the conceptual content of all items remained unchanged. Finally, the translation was judged by the Portuguese research team to be of high quality prior to data collection.

### 2.5. Data Analysis

Descriptive statistics were computed for participants’ characteristics and all scale variables, including frequencies, percentages, means, medians, standard deviations, skewness, and kurtosis. Based on the practices used in previous validation studies of the MHLq-YA and MHLq-SVa [1,27,29,30,31], a Confirmatory Factor Analysis (CFA) using the *lavaan* package in R 4.5.2 was initially conducted to examine whether the original four-factor structure could be replicated in the Greek sample.

The CFA used the Weighted Least Squares Mean and Variance Adjusted (WLSMV) estimator, which is appropriate for ordinal Likert-type and non-normal categorical indicators and for sample sizes above 200 [40]. Model fit was evaluated using the Comparative Fit Index (CFI), Tucker–Lewis Index (TLI), Root Mean Square Error of Approximation (RMSEA), and Standardized Root Mean Square Residual (SRMR) with its 95% confidence interval. Standardized factor loadings were examined to assess each item’s contribution to its respective latent factors. Based on theoretical considerations, residual covariances between selected item pairs were specified. Due to poor model fit indices, the research team proceeded—consistent with cross-cultural adaptation guidelines [41,42]—with an exploratory factor analysis to identify a structure better suited to the Greek data. The EFA was conducted in SPSS v.25, using principal axis factoring and oblique rotation, an extraction method that—unlike Maximum Likelihood extraction—does not assume multivariate normality. Items were removed based on both psychometric criteria (an a priori factor-loading cutoff of 0.40 and the fact that their removal did not improve reliability) and poor conceptual alignment with the emerging factor structure in the Greek. This approach is consistent with recommendations that item omission during EFA should be guided by theoretical coherence rather than driven solely by statistical thresholds [43].

Items with cross-loadings were retained on the factor that was conceptually most coherent when the difference between loadings was <0.10 and removal did not improve reliability. Internal consistency was assessed using Cronbach’s alpha. Convergent and discriminant validity were established through inter-scale and corrected item–total correlations and by correlating the MHLq-YA with MHPK-10 and MHSIS. Heterotrait–monotrait ratio of correlation (HTMT) ≤ 0.85 or 0.90 was also considered indicative of discriminant validity [44].

To evaluate the evidence of construct validity, known-group comparisons were conducted using independent-samples *t*-tests. We expected that women [27,28,45] and participants with education or experience in mental health (e.g., students in health/humanities fields, in higher semesters, who had attended mental health seminars, or knew someone with a mental health problem) would score higher on the MHLq-YA than their respective counterparts [28,30,46,47].

## 3. Results

### 3.1. Descriptives of the Items

The means of most items were close to the median, and standard deviations were less than half across all items. Nine out of the 25 items (Q1, Q9, Q11, Q20, Q25, Q26, Q13r, Q15r, Q5) showed kurtosis values above +2, indicating leptokurtic distributions with responses concentrated around the mean and more extreme values in the tails. Shapiro–Wilk tests indicated significant non-normality for all items (*p* < 0.001) (Table 2).

The average score for the 29 items is 115.62 (±12.10; range 29 to 145); for the ‘Knowledge of mental health problems’ factor 42.95 (±5.66; range 11 to 55); for the ‘Erroneous beliefs/stereotypes’ factor 32.93 (±3.98; range 8 to 40); for the ‘Help-seeking behavior and first aid skills’ 23.12 (±3.87; range 6 to 30); and for the’ Self-help strategies’ factor 16.62 (±2.47; range 4 to 20).

### 3.2. Construct Validity

Although the scaled indices of the four-factor model showed moderate model fit to the data [χ^2^(360) = 793.90, CFI = 0.880, TLI = 0.865, RMSEA = 0.076 (0.070–0.083), and SRMR = 0.095], the robust indices suggested substantially poorer fit [CFI = 0.669, TLI = 0.627, RMSEA = 0.117 (0.108–0.126)]. Standardized factor loadings ranged from 0.12 to 0.88, with particularly low loadings for Q4, Q10, Q12, Q18, and Q21. Several theoretically specified residual covariances were also included in the model. The CFA Model specification can be found in Supplementary S1.

Consequently, an exploratory factor analysis (Principal Axis Factoring with Oblimin rotation and Kaiser Normalization) was performed, yielding three factors with eigenvalues greater than 1. Sampling adequacy, which was established a priori (see Participants), was also supported by the Kaiser–Meyer–Olkin measure (KMO = 0.86), indicating a “meritorious” degree of sampling adequacy [36]. Bartlett’s Test of Sphericity (χ^2^(406) = 2237.7, *p* < 0.001) indicated that the correlation matrix was factorable and appropriate for factor analysis. The three factors explained 26.75%, 9.45%, and 5.72% of the variance, respectively, accounting for 41.93% of the total variance.

Compared with the original factor structure, four items (Q4, Q12, Q18, Q21r) were removed due to consistently weak loadings and poor conceptual alignment with the emerging factor structure [43]; two items also loaded on a different factor than in the English version (Q11 and Q14). The resulting 25-item solution comprised three factors. Four items from factor IV (‘Self-help strategies’) and two items from Factor III (‘Erroneous beliefs/stereotypes’) were incorporated into factor I (‘Knowledge of mental health problems’). Consequently, Factor I comprised sixteen Items (Q1, Q2, Q3, Q7, Q9, Q11, Q14, Q16, Q19, Q20, Q22, Q24, Q25, Q26, Q27, Q28), mostly reflecting “Knowledge of mental health issues and health behaviors”, and it was named accordingly. Factor II incorporated the remaining five reversed items (Q6, Q10, Q13, Q15, Q23) from the original Factor II (except those that loaded onto Factor I: Q11 and Q14). The items clearly reflect “Erroneous beliefs and stereotypes” (e.g., Q6 “Mental disorders don’t affect people’s behaviors”, Q13 “Mental disorders don’t affect people’s feelings”), so the name of Factor II remained unchanged. Factor III comprised four items (Q5, Q8, Q17, Q29) reflecting professional help-seeking behaviors, either for self or others, such as Q8 concerning self-help seeking from a psychologist and Q17 reflecting readiness to encourage a close person to seek psychiatric care. Consequently, Factor III appears to represent “Professional help-seeking behaviors” and was named accordingly. A full conceptual justification can be found in the Discussion Section. Factor loadings are presented in Table 3.

Known-group comparisons showed further evidence of construct validity. No significant differences in the total or factor scores across gender (men/women), department type (health/humanities/other), or attendance of mental health seminars (yes/no) were shown. Semester of undergraduate studies, however, differentiated scores on Factor II, with students in semesters higher than the second demonstrating higher scores than those in earlier semesters (22.20 ± 2.26 vs. 20.73 ± 3.25, *t*(171) = −3.811, *p* < 0.001), indicating fewer erroneous beliefs/stereotypes and, consequently, higher mental health literacy in this domain. Total score was significantly higher among students who reported knowing someone with a mental health problem compared to those who did not (103.96 ± 9.99 vs. 100.20 ± 13.66, *t*(171) = −2.090, *p* = 0.038). Factor 1 showed a marginally non-significant difference (65.31 ± 7.27 vs. 62.69 ± 10.30, *t*(171) = −1.964, *p* = 0.051). Full comparisons are provided in Supplementary S2.

The average score for the 25 items is 102.64 (SD = 11.21; range 25 to 125); for the “Knowledge of mental health issues and health behaviors” factor 64.46 (SD = 58.16; range 16 to 80); for the “Erroneous beliefs/stereotypes” factor 21.30 (SD = 3.08; range 5 to 25); and for the “Professional help-seeking behaviors” factor 16.88 (SD = 2.88; range 4 to 20).

### 3.3. Internal Consistency

The corrected item–total correlations ranged from 0.44 to 0.68 for the first factor, from 0.41 to 0.61 for the second factor, and from 0.54 to 0.67 for the third (Table 4). Removing items did not improve the internal consistency of any factor. The three MHLq-YA factors demonstrated satisfactory internal consistency, with alphas ranging from 0.70 to 0.89, and the overall score showed similarly high reliability (α = 0.84) (Table 5).

### 3.4. Convergent and Discriminant Validity

The MHLq-YA total score showed low positive correlations with the MHPK-10 total score. Factor I and the total score correlated above 0.20 with the MHPK-10, whereas Factors II and III demonstrated correlations below 0.20 (Table 5). Correlations with MHSIS were in the expected direction, with a moderately high positive association with the MHLq-YA Factor III (*r* = 0.56, *p* < 0.01), and weaker or non-significant associations with the other two factors (Table 5).

Inter-scale correlations were low to medium (Table 5). Factor I (“Knowledge for mental health issues and health behaviors”) correlated weakly with Factor II (“Erroneous beliefs/stereotypes”), moderately with Factor III (“professional help-seeking behaviors”), and strongly with the total score. Factor II correlated moderately with the total score and weakly with Factor III, while Factor III correlated strongly with the total score. HTMT values showed adequate discriminant validity, with clear separation between factors (HTMT = 0.03 for Factors 1–2, 0.73 for Factors 1–3, and 0.24 for Factors 2–3).

## 4. Discussion

The current study aimed to provide preliminary evidence of the psychometric properties of the 29-item version of the MHLq-YA in Greek. Most items showed means close to their medians and relatively small standard deviations, suggesting limited dispersion and generally symmetric response patterns. Nine items, however, exhibited leptokurtic distributions (kurtosis > +2), indicating concentrated responses around the central value, with a subset of participants endorsing extreme categories [42,49]. Although this pattern is consistent with findings from other studies [29] and it may reflect restricted variability for most respondents and potential polarization for a minority, the satisfactory α reliability, high corrected item–total correlations, and substantial factor loadings suggest that item discrimination was not compromised. Although all items were negatively skewed, this pattern does not necessarily indicate a ceiling effect; instead, it likely reflects the sample’s relatively high level of mental health literacy and fewer erroneous beliefs, particularly given that most participants were enrolled in Health and Humanities schools. This interpretation is consistent with findings from other studies [29]. Moreover, because robust estimation methods were used, the moderate deviations from normality did not adversely affect the analyses.

The four-factor structure of the original MHLq-YA was not replicated in the Greek university-student sample. This finding does not, however, demonstrate that the underlying theoretical model is invalid or that the observed differences necessarily reflect cultural differences. Alternative explanations include differences in sample characteristics, the university-student context, item functioning, translation or cultural interpretation of specific items, and the possibility that some items operate differently across populations. Methodologically, the CFA findings indicated that imposing the original four-factor structure on the present data was not appropriate and provided a basis for subsequently using EFA to explore whether an alternative factor solution could be identified. This sequence was consistent with the aim of this study, which was to conduct a preliminary psychometric evaluation rather than assume a priori that the original factor structure could be definitively reproduced.

The exploratory analysis supported a three-factor solution comprising 25 items allocated into ‘knowledge about mental health issues and health behaviors’, ‘erroneous beliefs and stereotypes’, and ‘professional help-seeking behaviors’. The three factors could be conceptually related to the broader components of mental health literacy described by Jorm [12]. Omitting four items to reach a better solution for the Greek data aligns with guidelines for cross-cultural scale adaptation, recognizing that certain items may not function equivalently across sociocultural settings [50]. This was the case in other validation studies too [31]. The solution therefore appears to preserve the multidimensional nature of mental health literacy while demonstrating a somewhat different structure in the Greek context. Because the three-factor structure was derived from the same sample, it should, however, be considered exploratory and provisional until independently replicated.

Although this structure explained 41.93% of the variance, such values are often common for multidimensional psychological measures, where several latent dimensions contribute to item responses. Therefore, the solution adequacy should be evaluated using a broader set of criteria (i.e., communalities, factor interpretability, loading pattern, residuals, replication, and theoretical coherence) rather than relying on a universal percentage cut-off [51]. Importantly, this structure explained a higher proportion of variance than the original version (41.93% vs. 36.9%) [27].

One notable difference concerned the integration of items from factor IV (Self-help strategies) with items from factor I (Knowledge of mental health problems). In the authors’ opinion, this may reflect the way students conceptualize health-promoting behaviors as part of their knowledge about maintaining or improving mental health (e.g., ‘Physical exercise contributes to good mental health’), so they were correctly classified as part of the ‘knowledge’ factor. Similarly, the placement of two items from Factor II concerning appropriate responses to people experiencing mental health difficulties (i.e., Q11: “If someone close to me had a mental disorder, I would listen to her/him without judging or criticizing” and Q14: “The sooner mental disorders are identified and treated, the better”) within the “knowledge” factor may indicate that these behaviors are conceptualized primarily as knowledge of appropriate responses rather than as erroneous beliefs or help-seeking behaviors. In contrast, Q12 (“Alcohol use may cause mental disorders”) was correctly excluded from Factor I, since the assertion that alcohol *causes* rather than co-occurs with mental disorders is inaccurate.

Another important difference was the emergence of the professional help-seeking factor. The retained items mostly concerned seeking or encouraging professional (psychological or psychiatric) help, either for self or others (e.g., Q8 concerning self-help seeking from a psychologist and Q17 reflecting readiness to encourage a close person to seek psychiatric care), respectively, suggesting that professional help-seeking constitutes a distinguishable behavioral component of mental health literacy in this sample. Items referring to help-seeking from non-professional sources such as relatives or friends (Q4 and 18) were rightfully excluded from Factor III. This factor captures the behavioral aspects of mental health literacy, including accurate help-seeking intentions and appropriate first-aid responses, and emerged as a conceptually distinct dimension in this sample.

The cross-loadings for two items further demonstrate the difficulty of distinctly allocating mental health literacy content to a single conceptual domain. Their final factor assignment was based jointly on statistical and conceptual criteria. Thus, Q5 (Factor III), although loading slightly higher on Factor I, was retained in Factor III because its content clearly reflects professional help-seeking behaviors (“If someone close to me had a mental disorder, I would encourage them to seek help from a psychologist”). Similarly, item Q11 (‘If someone close to me had a mental health issue, I would listen to them without judging or criticizing’), despite having a secondary loading on Factor III, was retained in Factor I because it represents knowledge of how to respond to people experiencing mental health issues. The strong coefficients, providing support for the internal consistency of the three factors, support these decisions and provide preliminary psychometric evidence for the adapted factor structure of the scale. In summary, although these shifts may reflect meaningful cultural or linguistic differences in how specific items are interpreted, rather than deficiencies in the scale, the observed differences should not be interpreted as definitive evidence of cultural differences in the construct itself. The divergence from the original factor structure may also reflect differences in translation, item interpretation, sampling characteristics, or the exploratory nature of the analysis. Further research using formal semantic-equivalence procedures, independent confirmatory factor analysis, measurement-invariance testing, and more diverse samples is therefore needed to determine whether the three-factor structure is robust and culturally specific. Despite these differences, the findings are consistent with previous multicultural studies, indicating generally high levels of mental health literacy among university students [1].

The known-group comparisons also provided some preliminary support for construct validity. Mental health literacy differed according to students’ educational stage and personal exposure to mental health problems, with students in later semesters tending to score higher on the relevant factor, while students who reported knowing someone with a mental health problem had higher overall mental health literacy. These differences align with previous research that greater education and personal experience are associated with greater mental health literacy [28,30,46,47]. However, the relatively limited number of significant group differences suggests that these findings should be interpreted cautiously. The overall reliability coefficients for MHLq-YA and its three factors were generally higher than those of the original version [27], indicating satisfactory internal consistency in the Greek context. Several properties related to the sample and setting may have reduced measurement error and enhanced internal consistency, such as improved item clarity through translation, the construct of health literacy being more culturally relevant for the specific population, and the sample being more homogeneous [52]. Additionally, improved reliability coefficients may result from the removal of poorly performing items [53].

The pattern of correlations provided preliminary evidence of convergent and discriminant validity. The small positive correlations with the MHPK-10 suggest that, although the two instruments measure related facets of mental health literacy, they emphasize different areas—MHPK-10 being exclusively knowledge-focused and MHLq-YA additionally capturing attitudinal and behavioral dimensions. Further support was provided by the moderately high, yet meaningful, correlation between the MHSIS and the MHLq-YA ‘Professional help-seeking behaviors’ factor, reflecting their theoretical alignment in assessing help-seeking orientations. The pattern of inter-factor correlations provided information about the degree of overlap among the dimensions. The relatively weak association between ‘knowledge’ and ‘erroneous beliefs/stereotypes’ factors suggests that these domains, although related, represent conceptually distinct aspects of mental health literacy. The moderate association between ‘knowledge’ and ‘professional help-seeking behaviors’ also reflects their expected theoretical relationship and suggests that students who are more informed also feel more capable of taking supportive action. We acknowledge that the homogeneous sample used in the present study may have limited the practical significance of these correlations. However, the preliminary evidence of discriminant validity was reinforced by low HTMT values suggesting adequate separation among the three factors, particularly for the ‘erroneous beliefs/stereotypes’ (Factor II). Together, these findings provide some support that mental health literacy among Greek university students is a multidimensional construct, with knowledge, beliefs, and self-help skills connected yet meaningfully differentiated.

This study has several limitations that should be acknowledged. First, the cross-sectional design and convenience–snowball sampling restrict the generalizability of the findings. Although the sample consisted predominantly of female and highly educated university students (e.g., bachelor’s-level education) and this pattern aligns with other validation studies [29,31,45] and with the study’s required age-restricted recruitment, it may not fully represent the broader population of young adults in Greece. Importantly, because four items from the original scale were omitted in the present study, the possibility that the emerging factor structure and item reduction were partly influenced by characteristics or potential biases of the sample (i.e., predominance of university students and the relatively modest and non-representative sample) cannot be excluded. Accordingly, the three-factor structure identified in this study should be considered specific to the present sample rather than definitively representative of the broader Greek young-adult population. Future studies should use larger, more heterogeneous, and preferably probability-based or stratified samples to examine the generalizability of the factor structure and determine whether the shortened item pool reflects genuine cultural differences that generalize to non-student young adults. Second, data were collected exclusively through online self-report measures. Although university students are generally expected to possess adequate digital skills, online administration may have introduced response biases and limited participation to individuals with sufficient internet access and digital literacy. Future research could therefore incorporate mixed-mode data collection (e.g., combining online and in-person administration). Third, although the sample size was sufficient for the exploratory factor analysis, it was relatively modest for more advanced psychometric modelling and was rather insufficient to establish stability and generalizability. Most importantly, the three-factor structure identified in this study was derived through exploratory factor analysis and, although its adequacy was examined through sampling adequacy and factorability, factor loadings and cross-loadings, explained variance, conceptual interpretability, and internal consistency, it was not subsequently cross-validated in an independent sample, which would be essential to determine the stability and replicability of the proposed structure. Furthermore, because the same sample was used to derive the structure, we cannot determine from the present study whether the three-factor solution would reproduce in an independent sample. Therefore, the three-factor structure does not confirm or definitively establish the model; rather, it should be considered provisional and its further confirmation in a larger and more heterogeneous Greek sample should be prioritised in future research. Future studies could further examine measurement invariance across gender and age groups, regions, or cultural subgroups. Fourth, although the translation and cultural adaptation process included think-aloud pilot testing and expert review, formal quantitative assessment of semantic equivalence was not conducted, and future research should incorporate these. Finally, several psychometric properties need to be examined. Test–retest reliability is needed to establish the temporal stability of the scale, while predictive and behavioral validity should be examined to determine whether mental health literacy relates to actual help-seeking behavior, stigma, or relevant mental health outcomes. These additional analyses would provide stronger evidence regarding the reliability, validity, and broader applicability of the Greek MHLq-YA.

## 5. Conclusions

Although the findings should be interpreted in light of the study’s limitations, this study provides preliminary psychometric evidence supporting the use of a provisionally adapted 25-item version of the MHLq-YA (MHLq-YA-GR) for assessing mental health literacy among Greek university students. The exploratory analysis yielded a provisional three-factor solution—knowledge about mental health issues and health behaviors, erroneous beliefs/stereotypes, and professional help-seeking behaviors—that are broadly consistent with Jorm’s conceptualization of mental health literacy [12]. Overall, the findings provided preliminary evidence of internal consistency and construct validity, while the factor structure requires confirmation in independent and more heterogeneous samples. The findings contribute to ongoing discussions about the dimensionality of mental health literacy and underscore the importance of culturally grounded validation rather than assuming universal factor structures.

## Figures and Tables

**Table 1:** Sample descriptive characteristics (*n* = 211).

Variables		*n* (%)
Sex	Women	147 (69.7)
	Men	63 (29.9)
Academic qualification	Bachelor’s degree	201 (95.3)
	Master’s degree	6 (2.8)
	PhD	1 (0.5)
	Other	3 (1.9)
Academic department	Health and humanities	119 (56.4)
	Engineering	14 (6.6)
	Natural Sciences	4 (1.9)
	Agricultural Sciences	14 (6.6)
	Management and Finances	58 (27.5)
	Music and audiovisual	1 (0.5)
Age		20.72 (SD 1.72, min. 19 max. 25)
Family status	Married	5 (2.4)
	Single	147 (69.7)
	Divorced	5 (2.4)
	In relationship	38 (18)
	Other	16 (7.6)
Nationality	Greek	209 (99.1)
	Bulgarian	1 (0.5)
	Romanian	1 (0.5)
Type of Region in Crete (Greece)	Urban	151 (71.6)
	Semi-urban	33 (15.6)
	Rural	18 (18.5)
	Other	5 (2.4)
Type of relationship With the person with Mental Health problem	Friend	49 (23.2)
	Relative	48 (22.7)
	Myself	13 (6.2)
	Other	29 (13.7)
Know someone with Mental Health problems	Yes	70 (33.2)
	No	103 (48.8)
I have myself mental health problems	Yes	13 (6.2)
	No	126 (59.7)
A relative has mental health problems	Yes	48 (22.7)
	No	91 (43.1)
Have you ever attended seminars for mental health	Yes	84 (39.8)
	No	127 (60.2)

**Table 2:** Item and total score descriptive characteristics and normality indices.

Items	Mean (SD)	Median	Skewness	Kurtosis	W
1	4.30 (0.84)	4.00	−1.64	3.60	0.728
2	3.81 (0.88)	4.00	−0.77	0.86	0.848
3	3.86 (0.75)	4.00	0.75	0.85	0.823
4	3.06 (1.16)	3.00	−0.08	−0.87	0.914
5	4.49 (0.80)	5.00	−1.93	4.21	0.658
6r	4.27 (0.89)	4.00	−0.1.36	1.87	0.761
7	4.16 (0.80)	4.00	−0.92	1.28	0.807
8	4.18 (0.92)	4.00	−0.99	0.52	0.800
9	4.15 (0.71)	4.00	−1.02	2.83	0.770
10r	3.99 (0.99)	4.00	−0.29	−1.16	0.826
11	4.30 (0.95)	5.00	−1.51	2.18	0.733
12	3.39 (0.99)	4.00	−0.40	−0.28	0.895
13r	4.29 (0.94)	5.00	−1.55	2.22	0.730
14	3.98 (0.88)	4.00	−0.517	−0.273	0.852
15r	4.50 (0.85)	5.00	−2.24	5.64	0.620
16	3.72 (0.84)	4.00	−261	0.031	0.865
17	4.17 (0.95)	4.00	−1.22	1.27	0.787
18	3.18 (1.10)	3.00	−0.34	−0.48	0.904
19	3.95 (0.83)	4.00	−0.861	1.29	0.828
20	4.27 (0.74)	4.00	−1.12	2.30	0.767
21r	3.35 (1.06)	3.00	−0.18	−0.49	0.908
22	3.62 (0.83)	4.00	−0.32	0.36	0.862
23r	4.26 (0.93)	5.00	−1.18	0.80	0.764
24	3.97 (0.90)	4.00	−0.86	0.90	0.839
25	4.25 (0.76)	4.00	−1.19	2.60	0.768
26	4.20 (0.85)	4.00	−1.33	2.55	0.774
27	4.05 (0.90)	4.00	−0.99	1.15	0.823
28	3.86 (0.84)	4.00	−0.82	1.11	0.833
29	4.04 (0.95)	4.00	−0.85	0.32	0.834
Total	115.62 (12.10)	116.00	−1.64	5.11	0.728

Note. W: Shapiro–Wilk. All 29 items show significant non-normality (*p* < 0.001); ‘r’ indicates that the items are reverse-scored.

**Table 3:** Exploratory factor analysis results (factor loadings, eigenvalues, variance explained), and descriptive statistics for the three factors (*n* = 211).

Items	Original Version	Factor 1 (Greek)	Factor 2 (Greek)	Factor 3 (Greek)	Status
20	F1	0.71 √			Retained—same factor
25	F1	0.69 √			Retained—same factor
27	F1	**0.69** √			Retained—same factor
26	F4	**0.67**		0.47	Reassigned: F4 → F1; cross-loading
19	F4	0.62			Reassigned: F4 → F1
3	F1	0.61 √			Retained—same factor
5	F3	0.59		**0.55** √	Retained—same factor; cross-loading
9	F1	0.58 √			Retained—same factor
24	F1	0.57 √			Retained—same factor
22	F1	0.57 √			Retained—same factor
28	F1	0.55 √		0.40	Retained—same factor; cross-loading
7	F4	0.54			Reassigned: F4 → F1
11	F2	**0.51**		0.44	Reassigned: F2 → F1; cross-loading
2	F1	0.51 √			Retained—same factor
16	F1	0.50 √			Retained—same factor
1	F4	0.48			Reassigned: F4 → F1
14	F2	0.48			Reassigned: F2 → F1
12	F1				Deleted
15r	F2		0.76 √		Retained—same factor
13r	F2		0.62 √		Retained—same factor
23r	F2		0.53 √		Retained—same factor
6r	F2		0.52 √		Retained—same factor
10r	F2		0.41 √		Retained—same factor
18	F3				Deleted
4	F3				Deleted
21r	F2				Deleted
29	F3			0.80 √	Retained—same factor
8	F3	0.41		0.74 √	Retained—same factor; cross-loading
17	F3	0.52		**0.65** √	Retained—same factor; cross-loading
Eigenvalues		7.76	2.73	1.66	
% of variance explained		26.75	9.45	5.72	
Cumulative percentage		26.75	36.19	41.93	
Score range		16–80	5–25	4–20	4–20
Mean (sd)		64.46 (8.16)	21.30 (3.08)	16.88 (2.88)	16.88 (2.88)
Std. Error of Mean		0.56	0.21	0.20	0.20
Median		65.00	22.00	17.00	17.00

Note. Exploratory factor analysis was performed using Principal Axis Factoring with Oblimin rotation and Kaiser Normalization. Factor loadings ≥ 0.40 are presented. F1, F2, F3, and F4 denote the factors to which each item pertains in the original version. √ symbol indicates that the item loaded in the same factor as in the original version. Bolded loadings denote the factor to which the item was ultimately assigned despite cross-loadings. Strikethrough values indicate loadings that were not retained in the specific factor.

**Table 4:** The Greek translation of MHLq-YA and associated reliability indices.

No	English Version	Greek Version	Cronbach’s Alpha if Item Deleted	Corrected Item–Total Correlations
Factor 1: Knowledge of mental health issues and health behaviors				
1	Physical exercise contributes to good mental health.	H φυσική (σωματική) άσκηση συμβάλλει στην καλή ψυχική υγεία	0.89	0.44
2	A person with depression feels very miserable.	Ένα άτομο με κατάθλιψη αισθάνεται δυστυχισμένο.	0.89	0.44
3	People with schizophrenia usually have delusions (e.g., they may believe they are constantly being followed and observed).	Άτομα με σχιζοφρένεια έχουν συνήθως ψευδαισθήσεις (π.χ νομίζουν ότι ακολουθούνται ή διώκονται συνεχώς από κάποιον ή κάτι)	0.88	0.56
7	Sleeping well contributes to a good mental health.	O καλός ύπνος συμβάλλει στην καλή ψυχική υγεία.	0.88	0.50
9	A person with anxiety disorder may panic in situations that she/he fears.	Ένα άτομο με αγχώδη διαταραχή μπορεί να παρουσιάσει πανικό σε καταστάσεις τις οποίες ήδη φοβάται.	0.88	0.55
11	If someone close to me had a mental disorder, I would listen to her/him without judging or criticising.	Εάν κάποιο κοντινό μου άτομο είχε κάποια ψυχική διαταραχή, θα το άκουγα χωρίς να το κρίνω.	0.88	0.50
14	The sooner the mental disorders are identified and treated, the better.	Όσο πιο έγκαιρη είναι η διάγνωση, τόσο πιο αποτελεσματική είναι η αντιμετώπιση.	0.89	0.46
16	Changes in brain function may lead to the onset of mental disorders.	Aλλαγές στην λειτουργία του εγκεφάλου μπορούν να οδηγήσουν στην εμφάνιση ψυχικών διαταραχών.	0.89	0.46
19	A balanced diet contributes to good mental health.	H ισορροπημένη διατροφή συμβάλει στην καλή ψυχική υγεία.	0.88	0.59
20	One of the symptoms of depression is the loss of interest or pleasure in most things.	Ένα από τα συμπτώματα της κατάθλιψης είναι η απώλεια ενδιαφέροντος ή ευχαρίστησης στα περισσότερα πράγματα.	0.88	0.68
22	The symptom’s length is one of the important criteria for the diagnosis of a mental disorder.	H διάρκεια των συμπτωμάτων είναι ένα από τα σημαντικότερα κριτήρια για τη διάγνωση μίας ψυχικής ασθένειας.	0.88	0.53
24	Drug addiction may cause mental disorders.	O εθισμός στα ναρκωτικά μπορεί να προκαλέσει ψυχικές διαταραχές.	0.88	0.53
25	Mental disorders affect people’s thoughts.	Oι ψυχικές διαταραχές μπορούν να επηρεάσουν την σκέψη των ατόμων.	0.88	0.66
26	Doing something enjoyable contributes to a good mental health.	Το να κάνει κάποιος πράγματα που απολαμβάνει συμβάλλει στην καλή ψυχική υγεία.	0.88	0.66
27	A person with schizophrenia may see and hear things that nobody else sees and hears.	Ένα άτομο με σχιζοφρένεια μπορεί να βλέπει και να ακούει πράγματα που άλλοι δεν βλέπουν και ακούν.	0.88	0.67
28	Highly stressful situations may cause mental disorders.	Oι πολύ αγχωτικές καταστάσεις μπορεί να προκαλέσουν ψυχικές διαταραχές.	0.88	0.53
Factor 2: Erroneous beliefs/stereotypes				
6r	Mental disorders don’t affect people’s behaviours.	Oι ψυχικές διαταραχές δεν επηρεάζουν την συμπεριφορά.	0.66	0.45
10r	People with mental disorders belong to	Άτομα με ψυχικές διαταραχές προέρχονται από οικογένειες	0.72	0.30
13r	Mental disorders don’t affect people’s feelings.	Oι ψυχικές διαταραχές δεν επηρεάζουν τα συναισθήματα.	0.61	0.56
15r	Only adults have mental disorders.	Μόνο οι ενήλικες μπορεί να πάσχουν από ψυχικές διαταραχές.	0.60	0.61
23r	Depression is not a true mental disorder.	H κατάθλιψη δεν είναι πραγματική ψυχική διαταραχή.	0.68	0.41
Factor 3: professional help-seeking behaviors				
5	If someone close to me had a mental disorder, I would encourage er/him to look for a psychologist.	Εάν κάποιο κοντινό μου άτομο είχε κάποια ψυχική διαταραχή, θα το ενθάρρυνα να απευθυνθεί σε έναν ψυχολόγο.	0.79	0.55
8	If I had a mental disorder I would seek for a psychologist’s help.	Εάν είχα κάποια ψυχική διαταραχή, θα αναζητούσα βοήθεια από έναν ψυχολόγο.	0.74	0.64
17	If someone close to me had a mental disorder, I would encourage her/him to see a psychiatrist.	Εάν κάποιο κοντινό μου άτομο είχε κάποια ψυχική διαταραχή, θα το ενθάρρυνα να απευθυνθεί σε κάποιο ψυχίατρο.	0.76	0.62
29	If I had a mental disorder I would seek for a psychiatrist’s help.	Εάν είχα κάποια ψυχική διαταραχή, θα αναζητούσα βοήθεια από έναν ψυχίατρο.	0.73	0.67

Note. The items of the scale were originally published in English and Portuguese [27].

**Table 5:** MHL-YA inter-factor correlations (shaded cells), Heterotrait–Monotrait ratio of correlations (HTMT), and reliability indices.

Factors	FI	FII	FIII	Total	Cronbach’s Alpha
FI		0.03	0.73		0.89 (95% CI [ 0.87, 0.91])
FII	0.23 **		0.24		0.70 (95% CI [ 0.63, 0.76])
FIII	0.61 **	0.17 *			0.80 (95% CI [ 0.75, 0.84])
Total	0.95 **	0.45 **	0.75 **		0.89 (95% CI [ 0.86, 0.91])
MHPK10	0.25 **	0.21 **	0.16 *	0.26 **	
MHSIS	0.26 **	0.09	0.56 **	0.36 **	0.77 (95% CI [ 0.68, 0.84])

Note. F1: “Knowledge for mental health issues and health behaviors”; F2: “Erroneous beliefs/stereotypes”; F3: “Professional help-seeking behaviors”. Correlations are presented in the shaded cells. HTMT values are presented in the corresponding three mirrored white cells in the upper section of the table. Cronbach’s α coefficients are reported with 95% confidence intervals (CIs), calculated using the Feldt method [48]. ** *p* < 0.01, * *p* < 0.05.

## Data Availability

The data presented in this study are available on request from the corresponding author due to privacy.

## Supplementary Materials

The following supporting information can be downloaded at https://www.mdpi.com/article/10.3390/healthcare14203366/s1. Supplementary S1: CFA lavaan Syntax; Supplementary S2: Differences in MHLq-YA scores according to sociodemographic characteristics and mental health-related experiences.
