# Supplementary material for: Preliminary Psychometric Evaluation of the Mental Health Literacy Questionnaire for Young Adults (MHLq-YA) Among Greek University Students
Source: Healthcare (Basel). 2026 Oct 9;14(20):3366. doi: 10.3390/healthcare14203366 (PMC13704357; doi:10.3390/healthcare14203366)
Supplement: Supplementary file 1 [file healthcare-14-03366-s001.zip › S1.pdf]

Supplementary S1. CFA lavaan Syntax

```
library (lavaan)
str(MHL_29)


inspect(fit.modindices)
options(max.print=9999999)


model <- '
MHL_1 =~ Q2 + Q3 + Q9 + Q12 + Q16 +Q20 + Q22 + Q24+ Q25 + Q27 + Q28
MHL_2 =~ Q6 + Q10 + Q11 + Q13 + Q14 + Q15 + Q21 + Q23
MHL_3 =~ Q4 + Q5 + Q8+ Q17+Q18 + Q29
MHL_4 =~ Q1 + Q7 + Q19 + Q26
Q2 ~~ Q13
Q2 ~~ Q18
Q3 ~~ Q28
Q12 ~~ Q24
Q12 ~~ Q28
Q12 ~~ Q24
Q12 ~~ Q28
Q11 ~~ Q14
Q15 ~~ Q19
Q4 ~~ Q18
Q5 ~~ Q29
Q8 ~~ Q29
Q7 ~~ Q19'
#factor 1 MHL_1
#factor 2 MHL_2
#factor 3 MHL_3
#factor 4 MHL_4

fit <- cfa(model, data=MHL_29_2_, estimator =
"WLSMV", ordered = c( "Q1", "Q2", "Q3", "Q4", "Q5", "Q6", "Q7", "Q8", "Q9", "Q10", "Q
11", "Q12", "Q13", "Q14", "Q15", "Q16", "Q17", "Q18", "Q19", "Q20", "Q21", "Q22", "Q23
", "Q24", "Q25", "Q26", "Q27", "Q28", "Q29"))

summary(fit,fit.measures=TRUE,standardized=TRUE)
```
