# Supplementary material for: Preliminary Psychometric Evaluation of the Mental Health Literacy Questionnaire for Young Adults (MHLq-YA) Among Greek University Students
Source: Healthcare (Basel). 2026 Oct 9;14(20):3366. doi: 10.3390/healthcare14203366 (PMC13704357; doi:10.3390/healthcare14203366)
Supplement: Supplementary file 1 [file healthcare-14-03366-s001.zip › S2.pdf]

Supplementary S2. Differences in MHLq-YA scores according to sociodemographic characteristics and mental health-related experiences

		**MHLq-YA Factor I**			**MHLq-YA Factor II**			**MHLq-YA Factor III**			**MHLq-YA Total I**		
**Variables**	**Categories**	M (SD)	*t*	*p*	M (SD)	*t*	*p*	M (SD)	*t*	*p*	M (SD)	*t*	*p*
**Gender**	Male	65.32 (8.25)	1.003	.317	21.38 (2.83)	.293	.769	16.62 (3.14)	-.910	.364	103.31 (11.29)	.577	.565
	Female	64.08 (8.14)			21.24 (3.17)			17.01 (2.75)			102.34 (11.24)		
**Semester (undergraduate students)**	Two first-years undergraduate	64.04 (8.08)	-0.600	.549	20.73 (3.25)	**-3.811**	**<.001**	16.87 (2.88)	-.152	.880	101.64 (10.94)	-1.399	.163
	Over 2 years of undergraduate	64.75 (8.36)			22.20 (2.26)			16.94 (3.10)			103.89 (11.43)		
**Type of Department**	Health and Humanities	64.86 (6.85)	.743	.458	1.29 (3.21)	.000	1.000	17.17 (2.67)	1.552	.122	103.31 (9.89)	.937	.350
	Other fields of study	64.01 (9.63)			1.28 (2.89)			16.55 (3.09)			101.84 (12.77)		
**Knowing someone with a mental health problem**	NO	62.69 (10.30)	-1.964	.051	21.01 (2.87)	-1.520	.130	16.50 (3.26)	-.896	.371	100.20 (13.66)	**-2.090**	**.038**
	YES	65.31 (7.27)			21.74 (3.20)			16.91 (2.76)			103.96 (9.99)		
**Attendance of mental health seminars**	NO	64.05 (8.20)	-.892	.373	21.06 (2.72)	-1.399	.163	16.85 (2.82)	-.193	.847	101.96 (11.10)	-1.082	.280
	YES	65.07 (8.10)			21.67 (3.53)			16.93 (2.97)			103.67 (11.35		
